# mRNA Therapeutic Modalities Design, Formulation and Manufacturing under Pharma 4.0 Principles

**DOI:** 10.3390/biomedicines10010050

**Published:** 2021-12-27

**Authors:** Andreas Ouranidis, Theofanis Vavilis, Evdokia Mandala, Christina Davidopoulou, Eleni Stamoula, Catherine K. Markopoulou, Anna Karagianni, Kyriakos Kachrimanis

**Affiliations:** 1 Department of Pharmaceutical Technology, School of Pharmacy, Aristotle University of Thessaloniki, 54124 Thessaloniki, Greece; cdavidof@pharm.auth.gr (C.D.); amarkopo@pharm.auth.gr (C.K.M.); karagiak@pharm.auth.gr (A.K.); kgk@pharm.auth.gr (K.K.); 2 Department of Chemical Engineering, Aristotle University of Thessaloniki, 54124 Thessaloniki, Greece; 3 Laboratory of Biology and Genetics, School of Medicine, Aristotle University of Thessaloniki, 54124 Thessaloniki, Greece; 4 Fourth Department of Internal Medicine, Aristotle University of Thessaloniki, 54124 Thessaloniki, Greece; emandala@auth.gr; 5 Department of Clinical Pharmacology, School of Medicine, Aristotle University of Thessaloniki, 54124 Thessaloniki, Greece; stamoula@auth.gr

**Keywords:** mRNA, lipid nanoparticle, nanomedicine, therapeutics, pharma industry 4.0, formulation, storage, CAR T-cell, protein replacement, mRNA vaccines

## Abstract

In the quest for a formidable weapon against the SARS-CoV-2 pandemic, mRNA therapeutics have stolen the spotlight. mRNA vaccines are a prime example of the benefits of mRNA approaches towards a broad array of clinical entities and druggable targets. Amongst these benefits is the rapid cycle “from design to production” of an mRNA product compared to their peptide counterparts, the mutability of the production line should another target be chosen, the side-stepping of safety issues posed by DNA therapeutics being permanently integrated into the transfected cell’s genome and the controlled precision over the translated peptides. Furthermore, mRNA applications are versatile: apart from vaccines it can be used as a replacement therapy, even to create chimeric antigen receptor T-cells or reprogram somatic cells. Still, the sudden global demand for mRNA has highlighted the shortcomings in its industrial production as well as its formulation, efficacy and applicability. Continuous, smart mRNA manufacturing 4.0 technologies have been recently proposed to address such challenges. In this work, we examine the lab and upscaled production of mRNA therapeutics, the mRNA modifications proposed that increase its efficacy and lower its immunogenicity, the vectors available for delivery and the stability considerations concerning long-term storage.

## 1. Introduction 

**Figure 1:**
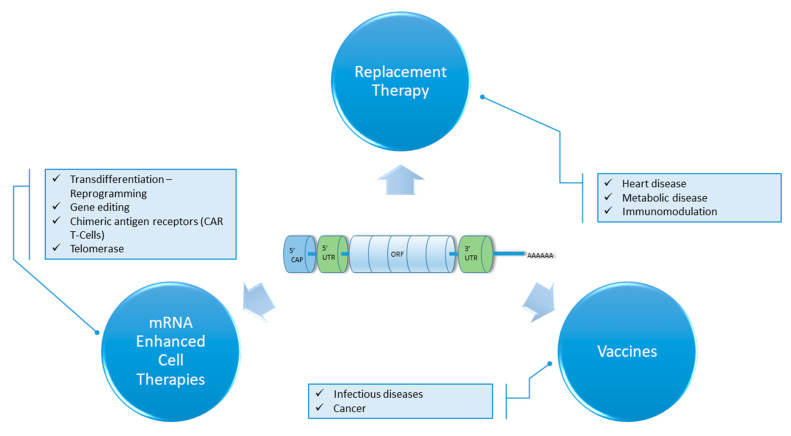
Main applications of mRNA in therapeutics broken down in the three major categories of replacement therapy, cell therapies and vaccines.

During recent decades, biopharmaceuticals, such as monoclonal antibodies, peptides or nucleic acids, have gained impressive attention as revolutionary therapeutics and vaccine strategies, attempting to fulfil the high expectations of resolving serious health conditions. Regarding nucleic acids, DNA- and mRNA-based technologies have been established as extremely promising approaches in therapy and prevention of numerous diseases, as part of so-called gene therapy. Specifically, the major technological and research innovations over the past decade have enabled mRNA to become a viable therapeutic solution, overcoming some of the associated challenges in its use, such as its short half-life and innate immunogenicity. mRNA’s great potential can manifest clinically through vaccination against infectious diseases and cancers, protein replacement therapies, tissue regeneration and the treatment of genetic diseases [1,2,3]. Furthermore, as mRNA technology matures, its convergence with emerging adoptive immunotherapies can materialize into safer and more effective approaches such as the development of state-of-the-art chimeric antigen receptor T-cells (Figure 1). The resulting cellular therapeutic product has several advantages compared to the one derived by a more classical approach, due to the transient nature of mRNA expression and non-integration of the foreign material to the cell’s genome: minimized cytotoxicity resulting from on-target off-tumor effects, easier production under good manufacturing practices favorable clinical validation and regulatory treatment [4,5,6,7,8].

From a structural point of view, mRNA presents itself as a single strand molecule which is transcribed from a DNA template and translated in functional proteins, thus playing a pivotal part in the central dogma of biology for flow of genetic information [9]. Mature mRNA in eukaryotic cells consists typically of five distinct structural elements: (a) 5′ cap—m7GpppN or m7Gp3N (N—any nucleotide), (b) 5′ untranslated region (5′UTR), (c) protein encoding open reading frame (ORF), starting with AUG codon, (d) 3′untranslated region (3′UTR) and (e) 100–250 adenosine-containing region (3′ poly(A)tail) [10]. Under physiological conditions, exogenous mRNA is translated by the ribosomal translation machinery and is naturally metabolized without integrating into the genome [11].

mRNA-based pharmaceuticals are considered advantageous over DNA-based ones for gene transfer and expression [12]. mRNA delivery vectors eliminate the requirement of both nuclear localization of the insert and the transcription step to introduce their functionality in cells. This way, mRNA can readily yield the desired protein instantly after being introduced into the cytoplasm of the cells, while at the same time its nature is ephemeral hence its degradation is orchestrated by numerous cellular mechanisms [13]. As such, mRNA-based technology involves no risk of random integration into host genomic DNA that could induce insertional mutagenesis or subsequent oncogenesis, while enabling rapid protein production [14]. mRNA can be tailored to have reduced immunogenicity, strong, adjustable expression concurrently with the enhanced safety that goes hand in hand with the properties mentioned. Furthermore, mRNA-based systems, can circumvent the limitations on transfection imposed by the cell cycle stage of target cells, displaying expression also in non-dividing cells, while the manipulation of cellular phenotype can occur in a more efficient, sophisticated and controllable manner [15,16,17]. Moreover, exogenous mRNA is manufactured in an easier and more cost-effective manner compared to peptides and cellular products originating from its use are not considered genetically modified organisms by regulatory affairs such as Food and Drug Administration [10]. However, the wide-spread use of mRNA as a therapeutic is hindered due to poor translatability in some cases, instability under long-term storage conditions, difficulties in manufacturing and up-scaling GMP compliant product, inefficient delivery and adverse immune reactions [18,19].

Accelerated development of mRNA-based technologies in vaccination and therapeutics throughout the last decades has resulted in several clinical and/or preclinical trials of many mRNA applications [20,21,22,23,24]. In the face of the global COVID-19 pandemic, many academic laboratories and their industrial partners are making a combined effort to resolve this health crisis by exploiting the promising mRNA vaccine platform. Given the urgency of the situation, an effective and safe vaccine that will become instantly available to the market in large amounts is of unprecedented need. At the time being, mRNA vaccines such as BNT162b2 from BioNTech and Pfizer and mRNA-1273 of Massachusetts-based Moderna have passed all clinical trials required and as such they are available at the market [25,26,27,28,29,30,31,32]. Moreover, their evaluation has moved forward in an astonishing rate, with studies examining the effect of booster doses, efficacy against different strains of the virus and different booster schemes. This commercial, clinical and regulatory success for mRNA products increases the global demand for an up-scaled production of efficacious and stable GMP grade in vitro transcribed (IVT) mRNA. Due to the fact that mRNA vaccines have never been mass-produced before, smaller biotechnological companies and institutions are collaborating with big biopharmaceutical companies for large-scale manufacturing and distribution. In May 2020, Moderna joined forces with Switzerland’s Lonza on a strategic collaboration planned to last ten years, with the aim of manufacturing up to 1 billion doses of mRNA-1273 annually, with the dose currently being at at 100 µg [33]. Serious efforts are therefore being made to increase the manufacturing capacity of mRNA-based products in order to meet global demand and improve the responsiveness to emerging and future outbreaks.

One of the most significant prerequisites in large-scale manufacturing of pharmaceutical products is the compliance of the production with the good manufacturing practices (GMP) or current good manufacturing practices (cGMP) conditions. These regulatory guidelines enact the minimum requirements that must be assured for consistent batch-to-batch manufacturing and quality control, in order to yield products of high quality and safety, appropriate to their intended use. GMP pharmaceutical development ensures that the quality attributes will meet the quality control standards that are necessary for the products to be tested in clinical trials and subsequent commercialization [34,35]. The inherent variability of biological agents coupled with the strict quality requirements poses a big technical challenge and overcoming this major challenge has garnered considerable attention from the scientific community and biotech industry [20,36]. To tackle this challenge, companies are trying to expand their facilities in order to meet the growing demands for clinical trials and product commercialization, such as CureVac, that have invested recently in considerable budget to commission a new GMP-qualified RNA production facility [37]. Once IVT mRNA GMP process manufacturing, with optimization, protocol validation and standardization, is accomplished, batch-to-batch reproducibility of highly pure, stable synthetic mRNA products, capable of supplying standard clinical trials and market demands, can be achieved [38]. Furthermore, such a process is flexible, highly scalable, easily standardized and can be carried out cost-effectively in a few weeks, including all the essential quality controls for a GMP production [39]. Sahin et al., estimated that the average cost of producing GMP batches of recombinant peptide therapeutics in a eukaryotic expression system is five to ten times higher than IVT mRNA, while Jackson et al., have currently stated that these processes allow GMP-compliant facilities to adjust to the production of a new vaccine within a very short period of time [25,38]. Moreover, continuous manufacturing transition is linked to improved quality control, material utilization, waste minimization and energy conservation when juxtaposed with similar biopharmaceutical batch operations. These advantages borne by process and automation control preached by the new pharma industry 4.0 paradigm become increasingly apparent as the demand for robust, reliable, productive and fast production lines becomes the new norm.

Pharma 4.0 industry will rationalize RNA nanosuspensions formulation and production by implementing Quality by Design (QbD) methodologies through particle informatics and in-silico process design [40,41]. These strategies will expand the design of experiment (DoE) approach by supplementing valuable insights into the causalities between formulation and process parameters and their dependencies with the critical quality attributes [42]. The latter shall inform the progress from descriptive to first principle approaches such as the realization of AI informed digital twins that aid with the system capacity upscaling and the seamless technology transfer from lab to industrial scale providing savings of raw materials, equipment, human and energy resources. Moreover, QbD is ideally applied by continuous pharmaceutical processes controlled by automation, robustness, reproducibility and real time validation. To this end Pharma 4.0 enabling tools of digital design such as spatial and charge arrangement formulation analysis, machine learning and systems-based simulations are empowering smart biomanufacturing strategies that pertain to unparalleled plant-wide quality assurance policies [43]. In addition, the thermodynamic stability and product performance of nanosuspensions may be predicted towards the assessment of solubility, predicting critical drug quality attributes linked to corresponding key process parameters also measured in real time by process analytical technology (PAT) [44].

In this review, we present an overview of all the steps taken for IVT mRNA, in both laboratory and industrial setting production, according to the latest advancements. Additionally, we provide a thorough review on the stability and immunogenicity improvement of mRNA-based technologies, the formulations and vectors used to deliver it, and finally storage conditions utilized to preserve the valuable finished product. In its entirety, the information presented here contributes to the realization of a continuous manufacturing line under 4.0 pharma principles, one able to produce stable and effective mRNA therapeutic products that meet the ever-increasing quality standards and global demand.

## 2. Basic Features of Moderna mRNA-1273 and Pfizer-BioNTech BNT162b2 COVID-19 mRNA Vaccines

In order to put into context the modifications and formulations presented throughout this review, it is useful to summarize characteristics of the two most thoroughly tested, widely employed and successful mRNA products on the market, the Moderna and BioNtech COVID-19 vaccines, which are liponanoparticle mRNAs. The Moderna mRNA product contains a 4004-nucleotide sequence encoding for the spike protein of the virus, whose features were not disclosed by the company but were retrieved through means of “reverse engineering” [45,46]. It features two serial proline substitutions at 986 and 987 aminoacid sites alongside the presence of the furin cleavage site, modifications that code for a stable prefusion S viral protein [47,48]. It is 5′ capped utilizing the cap 1 technology and its 5′ untranslated region is thought to be a patented V1-UTR [49]. The main coding sequence is characterized by substitution of uridine with N1-methylpseudouridine and codon sequence optimization that substitutes all GAA codons with GAG [46,48,50]. As a 3′ untranslated region, Moderna employs the one located on the human β-globin gene and terminates the sequence using three stop codons, while the poly-A tail remains to be determined [46]. The liponanoparticles contain 1,2- dimyristoyl-rac-glycero-3-methoxypolyethylene glycol-2000 (PEG2000-DMG), Heptadecan-9-yl 8-((2-hydroxyethyl)(8-(nonyloxy)− 8-oxooctyl)amino)octanoate)(SM-102),cholesterol and 1,2-distearoyl-sn-glycero-3-phosphocholine (DSPC). Excipients contained within the final carrier preparation include acetic acid, tromethamine and its hydrochloride salt, sucrose and sodium acetate [45]. It is dosed as 100 μg μRNA.

The mRNA of the BioNTech product has a length of 4284 nucleotides and it encodes the spike protein of the virus as well [45]. Like the Moderna vaccine, it features the same two serial proline substitutions but omits the furin cleavage site, resulting in a similar stable prefusion S protein. It is 5′ capped with a modified cap 1 analogue and the 5′ untranslated region is comprised by a fragment on the human α-globin gene [28,45]. The main coding sequence employs the same uridine substitutions for the modified analogues but compared to Moderna, utilizing a more lenient approach as far as GAA codons are concerned, leaving 14 of them unchanged [46]. The 3′ untranslated region of the construct features a hybrid sequence comprised of Amino-terminal enhancer of split gene sequence and mtRNR1 mitochondrial 12S ribosomal RNA 3′ regions, while it terminates with two UGA stop codons and features a segmented poly-A tail [46,51]. Nanoliposomes are formed utilizing DSPC, (4-hydroxybutyl)azanediyl)bis(hexane-6,1-diyl)bis(2-hexyldecanoate) (ALC-0315), cholesterol, and 2[(polyethylene glycol)-2000]-N,N-ditetradecylacetamide, while the excipients include potassium chloride, sodium chloride, sucrose, monobasic potassium phosphate and dibasic sodium phosphate dehydrate [45]. The finished product has a dosage of 30 μg mRNA.

## 3. mRNA Preparation Process

The starting point of an mRNA manufacturing process is considered to be the synthesis of the mRNA molecule. Typically, mRNA can be synthesized in the laboratory utilizing chemical and enzymatic methods. Baronti el al. have reviewed the most suitable methods for RNA preparation, presenting their advantages, disadvantages and their latest advances [52]. Chemical synthesis of mRNA displays an upper size chain limit of approximately 100 nucleotides after repetitive yields of >99%, while physiological mRNAs are typically 1000–10,000 nucleotides. While mRNA’s length is restrictive for its chemical synthesis, enzymatic in vitro transcription (IVT) is considered a simple and inexpensive procedure for mRNA synthesis that can yield products of variable sizes in milligram quantities [38,53].

In a commercial manufacturing process, sufficient amounts of functional pharmaceutical mRNA are typically synthesized in a cell-free system by in vitro transcription (IVT) of a DNA template encoding the protein of interest i.e., therapeutic protein or antigen of vaccine. The DNA template is preferably a plasmid DNA (pDNA) into which the gene of interest and an upstream RNA polymerase promoter have been inserted [54]. However, DNA template can be also a PCR product consisting of a promoter at the 5′ end or even two annealed oligonucleotides [55].

The most widely used template, the plasmid DNA, is isolated and purified from bacterial cells, e.g., *E. coli*, that they are cultured for the purpose of plasmid cloning. Cloning vectors of plasmid origin come with incorporated promoters facilitating in-vitro transcription, upstream of multiple cloning sites (MCS). Furthermore, poly A tail sequence, 5′ and 3′ UTR, and linearization restriction sites are also included at the pDNA template [56]. Currently, the in vitro transcription method using a highly processive bacteriophage RNA single-subunit polymerase (e.g., T7, T3, and SP6) has been widely established, as it is considered a cost-effective and easily scalable mRNA manufacturing process [38,57]. Bacteriophage T3, T7, and SP6 RNA polymerases are single polypeptide chains that require only Mg^2+^ as a cofactor and run off the DNA template after several transcription reactions [52]. Utilizing this process, high quantities of mRNA can be produced in a few hours, while through technical optimizations its feasibility has been dramatically improved. 

Pascolo et al. elucidated the specifics of pharmaceutical grade mRNA production with an IVT run off reaction of a plasmid DNA template (pDNA) [58]. The DNA manufacturing process includes several steps such as the production of synthetic DNA, plasmid cloning, plasmid purification, sequencing and validation of the DNA constructs or the insert, purification and characterization. After the desired DNA template has been obtained, the production method consists of the distinct mRNA preparation. To follow, we present the necessary steps of the commonly applied IVT mRNA preparation process, presented below in more detail [59,60,61].

### 3.1. Plasmid Linearization

In order to achieve plasmid linearization, the pDNA is treated in suitable conditions with an appropriate restriction enzyme. The specific recognition site for a restriction endonuclease is situated after the encoded poly(A) tail, if such exists. After the linearization, the restriction digest is terminated by adding suitable reagents e.g., EDTA, sodium acetate, proteinase K and the reaction is incubated in order to deactivate and remove the restriction enzyme. Purification of the linearized pDNA template ensues, usually by means of silica columns or chromatography, prior its use as an in-vitro transcription template. Other methods can also be utilized such as phenol extraction, followed by ethanol precipitation or centrifugal ultrafiltration, while there are commercial kits also available, such as Wizard^®^ DNA Clean-Up System and High Pure PCR purification kit. The purified sample consisting of the linearized pDNA is re-suspended in a buffer. A small sample of the DNA can be run on an agarose gel to ensure the linearization of the plasmid.

At the time of writing, plasmid linearization prior to in-vitro transcription is the dominant practice, adding to the cost and complexity of mRNA manufacturing. This might seem counterintuitive at first, given that there are naturally occurring sequences that can terminate transcription when encountered by the transcription enzyme [62]. Yet, it can be explained if one takes into account that enzymes utilized for transcription such as T7, have the propensity to read-through plasmid transcription termination signal. If the template plasmid is circular, this can yield a pool of mixed mRNA species since there is a probability that the enzyme will transcribe sequences residing downstream the desired sequence. On the other hand, if the plasmid is linearized, the enzyme will inevitably terminate transcription by running off the template’s edge. Given the applicability of an IVT system that could utilize circular plasmids without a prior linearization step, efforts have been made recently to engineer efficient transcription terminator sequences [63,64]. Such endeavors have already yielded sequences that can have up to 91% termination efficacy in vitro and further improvements could possibly herald a simpler mRNA production workflow [63].

### 3.2. In-Vitro mRNA Transcription

The linearized pDNA template containing an RNA polymerase site is typically treated with commercial kits, which include RNA polymerase and nucleotides triphosphates (NTPs) mix for mRNA synthesis. In general, the reaction takes place in a tube with inert and non-adsorptive walls (silicone or Teflon) that hosts the DNA template, a transcription buffer (e.g., HEPES or Tris HCl; NaCl, magnesium, dithiothreitol (DTT) and/or spermidine), natural or unnatural (modified) NTPs, an RNase inhibitor and an RNA polymerase. As has been mentioned, the polymerase may be a phage RNA polymerase (SP6, T3 or T7 RNA polymerases are common choices), and/or mutant polymerases such as, polymerases able to incorporate modified nucleic acids [65,66]. The reaction is incubated in suitable conditions, under constant mixing at 37 °C. Working area and pipettes are treated with an RNase decontamination solution. Then, typically, a DNase is added, mixed well and incubated in order to immediately remove the template pDNA enzymatically. In order to reduce enzyme utilization costs and abolish the generation of DNA fragments that can hybridize with the produced mRNA, plasmids, linearized or circular, can be removed via means of chromatographic techniques. Monolith columns, which are durable enough to be of industrial use, have been developed that can selectively retain and isolate mRNA or pDNA from complex mixtures [67,68,69]. More information on analytical approaches can be found in Section 4 of the current review.

In addition, if the IVT mRNA is lacking a 3′ poly(A) tail due to a “tailless” pDNA template, a further poly(A) tailing step is conducted post-transcriptionally by using a commercial poly(A)-tailing kit, containing poly(A) polymerase and an appropriate reaction buffer. Poly(A) tails of about 80 nucleotides and 160 nucleotides are considered functional and require about a 30-min tailing reaction. Due to concerns regarding production of mixed mRNA species with variable tail lengths, a putative problem that is discussed later on, it is suggested that the sequence giving the poly(A) tail is incorporated in the initial plasmid to be transcribed [70,71].

### 3.3. mRNA Purification

The purification of in-vitro transcribed mRNA is an essential step in any upstream manufacturing process, and it is included in all the preparation protocols. Impurities present in a non-ultra-pure clinically grade mRNA product can have serious impact on its translatability, function, quantification, batch-to-batch variability and quality standards. Moreover, contaminants as dsRNA, carry a sizable risk of immunogenicity that leads to unwanted activation of the cellular innate immune response and rapid degradation of the mRNA [19,71]. Purification can be achieved by several methods that can efficiently remove contaminants of the starting material or a by-product from the final mRNA transcript. Impurities typically include organic solvents, salts, free NTPs, cap analogs, protein impurities (such as restriction enzymes used, polymerases, DNAses or inhibitors of RNAses.), DNA-RNA hybrids or their fragments, bacterial genomic DNA contamination, residual DNA, short, truncated mRNAs and double stranded (ds)RNAs. The choice of the purification technique differs depending on whether they are to be employed on a small or on an industrial scale and as such they are discussed further on.

### 3.4. mRNA Encapsulation

Purified mRNA is rarely employed in its “naked” form, usually a delivery vector is used that protects and delivers the nucleic acid to the target cells [72]. The most commonly employed vectors, which are also the vectors chosen for the SARS-CoV-2 vaccines in circulation, are the mRNA-lipid nanoparticles (mRNA-LNPs) [73]. LNPs can be considered descendants of liposomes. Lipid nanoparticles differ from liposomes in two ways: lipid composition, where nanoparticles employ mostly solid lipids and architecture where liposomes feature an aqueous-filled cavity within a lipid bilayer [74]. Liposomes are prepared by forming a thin film of an organic solvent containing the desired lipids and after drying it, rehydrating it with a buffered aqueous solution containing the agent to be encapsulated [75,76]. This method yields a population of nucleic acid inclusions characterized by heterogeneous size usually exceeding 100 nm and low encapsulating yield that calls for supplementary extrusion or sonication techniques in order to achieve finer particle formation [77,78]. Another technique used is the ultrasonic-aided formation of a reverse emulsion (where water is trapped in oil) between an organic phase containing the encapsulating lipids and the payload in an aqueous phase, and low-pressure removal of the organic solvent [79].

For the production of mRNA-LNPs novel methods have been developed, namely crossflow/ethanol injection and microfluidic approaches [80,81,82]. During the ethanol injection procedure, the desired lipids (cationic lipids, cholesterol, “stealth” and “helper” lipids) are dissolved in ethanol, while the mRNA payload is dissolved in an aqueous buffer of acidic pH. Upon mixing, the cationic lipids become protonated due to the pH and form inverted micelles on mRNA, forming a “nucleation core” on which other lipids will be deposited upon [83,84]. Next, a dialysis step is performed to restore pH to the desired level. Apart from the lipid composition of the ethanol mixture, size of the LNPs is influenced by the rate of mixing, where a rapid rate results in smaller particles [80]. Microfluidic approaches allow precise adjustments in flow rates and subsequent mixing hence providing control over the size and guaranteed uniformity of the finished mRNA-LNPs [84].

## 4. Analytical Approaches

Analytical methods are the cornerstone of industrial production of both small molecule and advanced biopharmaceuticals. They constitute an integral part of the mandatory quality control as well as the isolation of high purity intermediates required for products whose synthesis is performed serially. It must be highlighted that in mRNA production, utmost purity of the final product is not only a safety issue, but also a matter of therapeutical efficacy and shelf-life longevity, since contaminants can compromise both shelf-life of the finalized product as well as illicit unwanted immune responses [85,86,87] 

### 4.1. For mRNA 

As already explained, the messenger RNA that is manufactured for a vaccine or therapy is produced through a cell-free process known as "in vitro transcription (IVT) synthesis". After mRNA has been IVT-synthesized, it is processed by enzymes that add a guanine nucleotide to its 5′ end (5′ cap), and a chain of several adenine nucleotides to its 3′ end. These post-transcriptional modifications are considered critical quality attributes as they protect the IVT-synthesized mRNA from degradation [20]. In the development of mRNA-based therapeutics and vaccines, LC and LC-MS procedures are used to ensure proper addition of the 5′ cap and poly-A tail, as well as to measure capping efficiency. Thus, Beverly and co-workers devised a method utilizing specific RNase H probes to cleave off 50 bases from the 5′ end of the mRNA. With a biotin-streptavidin-enriched sample, they were then able to perform LC-MS analysis of the cleaved fragments [88].

In another study in 2018, Beverly and colleagues combined an RNase digestion with poly-dT affinity enrichment to collect poly-A tail oligonucleotides from an mRNA and then analyzed the sample by LC-MS [89]. A recently introduced LC Hybrid Surface Technology (HST) seems to improve the quantification of low-level impurities. HST method for column and LC systems hardware, addresses and prevents nonspecific adsorption of analytes, particularly acidic compounds, to the electron-deficient metal surfaces of the LC fluid path. A highly cross-linked, ethylene-bridged siloxane polymer acts as a barrier that prevents analyte interactions with the metal surface [90]. In addition, in 2021 Muthmann and co-authors performed digestion of the entire transcript as initiated by nuclease P1, snake venom phosphodiesterase, and dephosphorylation. Finally, they generated single nucleosides with or without the cap and quantified these products with triple quadrupole mass spectrometry [91].

### 4.2. For the LPNs of mRNA

LNPs are the preferred delivery vessel for mRNA therapies and vaccines. These lipids include an ionizable, cationic lipid (such as MC3), a zwitterionic phospholipid (such as DSPC), cholesterol, and a pegylated lipid (such as PEG-DMG) [6].

In contrast to RNA, one additional challenge for lipids analysis is that most of lipids lack chromophores that might facilitate the use of the standard UV-vis detector conjugated with LC. Thus, a universal for HPLC detector, such as evaporative light scattering detector (ELSD) or charged aerosol detector (CAD) is proposed. Compared to the ELSD, the CAD has been demonstrated to offer, in lipid analysis, a more uniform response curve over a wider range of concentrations (over 4 orders of magnitude) with better accuracy, precision and lower limits of detections [92,93,94]

Such assays were used through stages of development as well as batch release. Primary work suggests that a Waters™ ACQUITY™ system, with low-adsorption hybrid surfaces might prove useful to establish more robust and accurate (improved recovery) methods [95] Similar study, shown that columns packed with a charged surface hybrid stationary phase provide advantageous selectivity and peak shape by using formic acid/formate-based mobile phases and evaporative light-scattering detection [96]. Advances in biotechnology require the development of state-of-the-art analytical techniques to ensure that LNP-encapsulated mRNAs are QC-labeled and tested to ensure their safety and efficacy. Modern techniques including liquid (LC), or gas chromatography (GC) and mass spectrometry (MS) are therefore being applied to analyze lipid and mRNA attributes or to detect and quantify lipid impurities [86].

### 4.3. For DNA Plasmid Purification

The recently discovered plasmid DNA vaccines, have gained tremendous attentions in the last few decades as a modern approach of vaccination. However, the purification of the pDNA vaccines is a crucial step in their production and administration, which is usually conducted by different chromatographic techniques and may require more than a single step to obtain the desired result [97]. Moreover, the purification of pDNA by chromatography may be confronted with other encounters, like the limited number of the existing stationary phases that have the capacity and ability to bind to the large biomolecules like pDNA [59].

Furthermore, soft beads packed columns may be susceptible to destroy as well as mass to transfer difficulties. These limitations led the researchers to evolve monolith columns for industrial use [98].

Recent chromatographic procedures related to purification of pDNA vaccines include anion exchange chromatography, affinity chromatography, multimodal chromatography, size exclusion and hydrophobic interaction chromatography [97,99,100,101,102].

### 4.4. For DNA Analysis

Currently, most of the DNA analysis methods are semi-quantitative, including polymerase chain reaction (PCR), sequencing, DNA chip and biosensors. Most of them rely on the calibration curves or the comparing threshold value comparison. However, for DNA reference material development, basic research of quantification methods was needed, in order to study the consistency of these methods and analyse the uncertainty sources [103]. UV spectrophotometry (UV) is commonly used for convenient DNA routine quantification by measuring the absorbance at 260 nm (OD260) based on Beer–Lambert’s law for high concentration and pure DNA samples. Some other physicochemical methods have high sensitivity, accuracy and clear metrology traceability; however, they are still often hampered by the efficiency of the phosphodiesterase enzyme digestion [104,105]. HR-ICPMS has higher sensitivity and specificity, which can accurately analyse the mass fraction of phosphorus, that stoichiometrically presents in DNA molecule and consequently achieve the DNA concentration with high precision and clear traceability [106].

However, a disadvantage of UV and HR-ICP-MS is their incapability of specifically distinguishing different DNA sequences. Thus, more advantageous techniques could be applied, as high resolution inductively coupled plasma mass spectrometry (HR-ICP-MS) to quantify the purified plasmids by analyzing the phosphorus in DNA molecules [107].

## 5. Differences between Laboratory and Industrial Scale

Differences in lab or clinical trial scale production and industrial production of mRNA products vary, in terms of logistics and technical feasibility as commanded by the increased production demands of the latter. The unprecedented demand for viable mRNA products brought forth by the recent pandemic has highlighted those differences in a tangible manner. Starting from IVT of mRNA, lab scale and clinical trial preparation of mRNA products allows for use of techniques that in an industrial setting could prove to be bottlenecks to the whole procedure: the small scale of the former can justify use of expensive reagents or reagents in short supply, such as modified nucleosides, enzymes or CleanCap reagent, that when scaled up could add prohibitively to the cost or procurement would not be possible due to short supply of GMP quality material [20,108]. Use of commercial all-in-one kits which streamlines small scale production is a common practice in laboratories which unfortunately is not an option for an industrial setting [109].

It is not only the upstream processes where disparities arise. In a recent review by Rosa et al., the importance of differences in downstream procedures are also made evident. In short, in the laboratory niche or small-scale development the fresh transcribed mRNA is cleaned and isolated from material that could increase immunogenicity or instability of the product, via DNase I digestion of the template and precipitation from the reaction’s solution using lithium chloride [19,110]. Still, those procedures fail to completely remove aberrant mRNA species and double stranded DNA, which makes the use of advanced chromatography techniques a one-way road for the industry [111].

**Figure 2:**
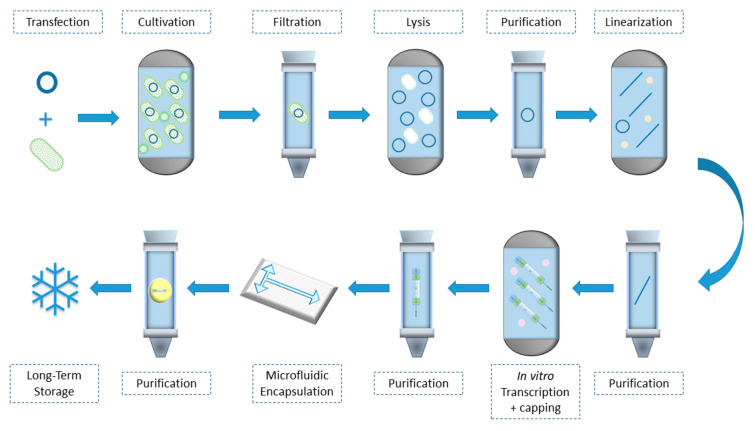
Production of mRNA on an industrial scale starting from bacterial transfection with the vector containing the DNA from which mRNA will be synthesized, down to its formulation with a delivery vector and long-term storage.

Ion pair reverse phase chromatography (IPC), ion exchange chromatography (IEC), diafiltration using tangential flow filtration (TFF) and affinity chromatography are some of the methods used to achieve pharmaceutical grade mRNA purity [19]. While all of them can theoretically be used, each of them has its drawbacks. IPC which captures mRNAs by the sugar-phosphate backbone can be expensive to scale up and makes use of toxic solvents like acetonitrile for the elution of the products. On the other hand, whereas IEC, a method that distinguishes molecule on basis of charge difference, is scalable and relatively inexpensive, it uses denaturing condition to achieve separation, adding to the complexity of the isolation the factor of precise temperature regulation [52,112]. Affinity chromatography manages to isolate the desired mRNA by taking advantage of its poly(A) tail, binding it on elements carrying deoxythymidine stretches and can achieve exceptionally high purity of product with the tradeoff of being less cost effective and being easily saturated, limiting its capacity per run [113,114]. TFF is a fast, economical and efficient procedure that can concomitantly diafilter and concentrate, providing a desalted and appropriately buffered product although it requires a denaturation and digestion workup to remove proteins and DNA [115,116]. The overview of the procedures is presented in Figure 2.

**Figure 3:**
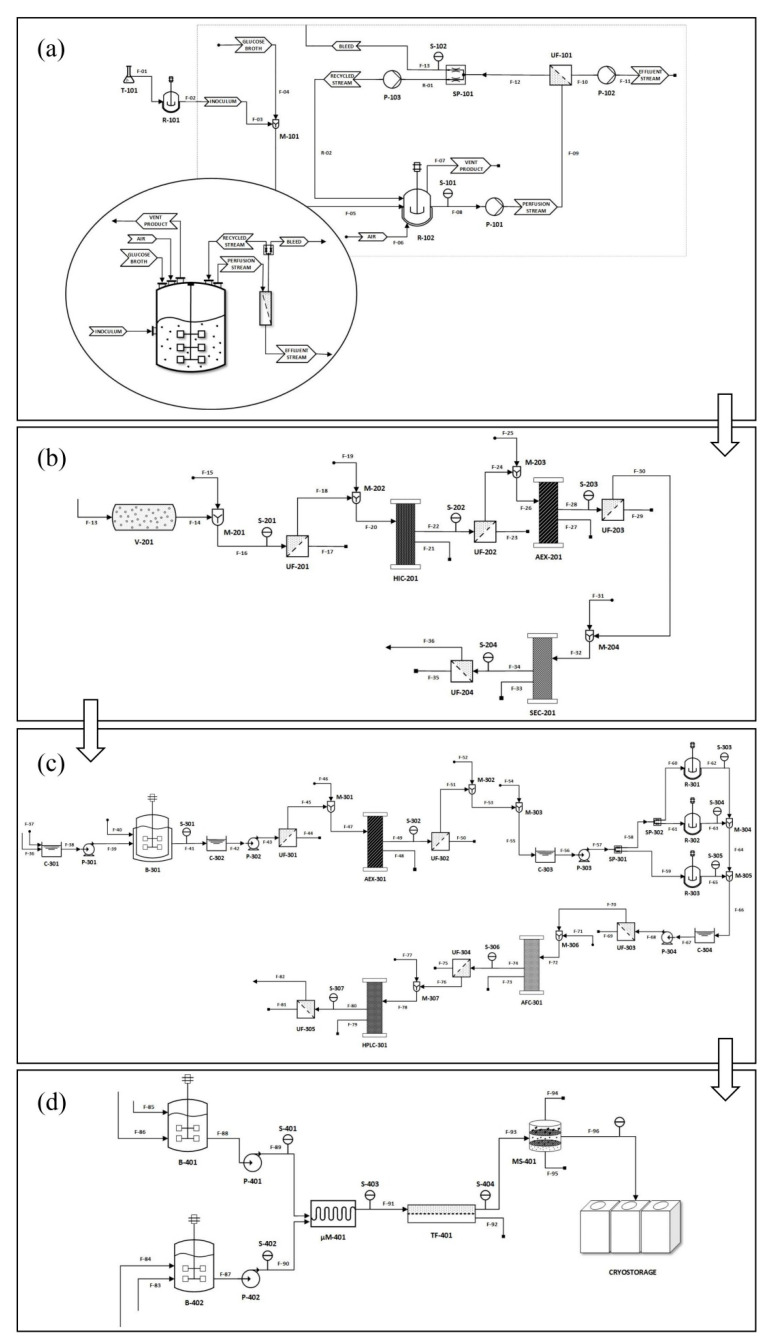
Process flow diagram of the Pharma 4.0, GMP compliant mRNA production (**a**) perfusion bacterial culture upstream; (**b**) cell lysis and plasmid purification; (**c**) linearization and in vitro transcription; (**d**) lipid nanoparticle microfluidic formulation [43].

In their review Rosa et al. also highlight the need for the development of a continuous, scalable procedure that can minimize costs, adumbrating its layout from the IVT reaction towards the TFF filtration of the product. While such a system has not yet been put into practice, a recent publication by Ouranidis et al. analyzes in silico and proposes scalable, continuous, end-to-end cGMP manufacturing processes that encompass all steps from plasmid-containing bacterial cultivation to final mRNA LNP formation (see Figure 3) [43]. The system utilizes a continuous perfusion bioreactor for bacterial cultivation (Figure 3a) which is also known as the upstream bioprocessing stage, namely the stage in which the product is being produced. The upstream processing is followed by the downstream bioprocessing stages (Figure 3b–d) in which the product is going through some modifications until it takes its final form. The first downstream processing includes the alkaline lysis of the *E. coli* cells to release the plasmid DNA stored in their core and the various chromatographic steps to harvest and purify the p-DNA (Figure 3b).

The purified p-DNA afterwards becomes linear using the EcoRI restriction enzyme [117] in order to go under an in-vitro transcription procedure and produce the corresponding mRNA (Figure 3c). The transcription takes place with the help of a T7 phage polymerase and capping is implemented by the utilization of vaccinia virus enzymes, under conditions that inhibit dsRNA formation. Finally, in the last downstream processing step (Figure 3d), the mRNA is purified by affinity chromatography utilizing a poly(U)-Sepharose/Oligo(dT) cellulose resin and encapsulation in lipid nanoparticles is achieved by microfluidic mixing of the pure mRNA with cationic lipids. The proposed process flow design emancipates an automated, continuous GMP compliant production, inline with pharma 4.0 principles of standardization, delivering roughly 270 million 30 μg mRNA doses per year.

## 6. Formulation Strategies

A cornucopia of different strategies is available to enhance the mRNA cellular uptake both under in vitro conditions but also in vivo. All of them intend to protect mRNA from degradation, shield its negative charge and provide an efficient platform for its intracellular delivery [118]. In the following section, some of the most significant formulation strategies for the mRNA delivery are discussed.

### 6.1. Viral Vectors

Very often, the formulation strategies for mRNA delivery are categorized in terms of the use of a viral or a non-viral vector. Viral vectors avail genetically-modified viruses as carriers of the genetic information. Commonly used viruses include retrovirus-derived vectors, adeno-associated viruses (AAV), alphaviruses, picornaviruses and flavivirus, which have substituted parts or all of the viral genome with the desired therapeutic or marker gene [119]. The preferable positive-sense RNA viruses have RNA genome that is able to be replicated or translated by the host ribosomes directly in the cytoplasm, producing large amounts of proteins [17]. However, viral vectors have difficulties in their construction, scale-up production and precise control of the gene expression, while there are safety issues to be concerned. They are subjected to significant limitations concerning immunogenicity and toxicity [120]. Although a lot of efforts have been made in order to eliminate these challenges, e.g., by using mutant vectors, self-inactivating vectors, nonsegmented negative-stranded (NSNS) viruses, such as Sendai virus (SeV) [119,121,122], the superiority of non-viral vectors for mRNA delivery is still integral [12,123].

### 6.2. Non-Viral Vectors

Non-viral vectors exploiting advances of nanotechnology platforms are in the focus of current research activities [124]. Non-viral vectors can be further classified into lipid-based systems solely consisting of lipid carriers, systems based on polymers, and hybrid systems consisting of both lipid and polymer chemical species [125,126].

#### 6.2.1. Polymer-Based Vectors

Among the non-viral vectors, polymer-based vectors have gained great attention and are commonly used as delivery systems for mRNA-based pharmaceutics. These systems are based on nanoparticles (NPs) consisting mainly of cationic polymers that favor electrostatic interactions with the negatively charged mRNA and the cell membrane, facilitating both the encapsulation and the cellular uptake and are typically manufactured via nanoprecipitation or emulsion techniques [127,128,129]. The employed cationic polymers should be biocompatible, free of toxicity as well as unwanted immunogenic effects. One important benefit of polymers application is their chemical flexibility and their capability to be surface-modified, facilitating the targeting and controlling the release model as through pH-responsive release [130]. The typical polymers possess positively charged amino groups and can be either synthetic, such as poly-L-lysine (PLL), polyamidoamine (PAMAM), poly(acrylic acid) (PAA) and polyethyleneimine (PEI), or natural, like chitosan [131]. PEI and its derivatives are very popular cationic polymers, due to their high cationic charge density and high transfection efficiency [132]. However, polymeric materials, such as PEI have presented toxicity issues, related to high molecular weight, polydispersity and clearance or biodegradation [118,133]. For this reason, several modification approaches have been tried, fabricating indicatively grafted salicylamide with PEI [134], graphene oxide (GO)- PEI complexes (CHOI), micelles of conjugated PEI-2k with stearic acid [135], conjugated PEI with cyclodextrin [136], reducible polycations with histidine and polylysine residues (HIS-RPCs) [137], poly(glycoamidoamine) brushes produced from poly(glycoamidoamine) and epoxides etc. Poly(lactic-co-glycolic acid) (PLGA) nanoparticles have also been utilized for mRNA delivery, but with low encapsulation efficiency [138].

However, the biggest effort of the researchers is on the investigation and synthesis of novel, biodegradable polymers or block co-polymers, specifically able to aid the efficient delivery of nucleic acids [139,140,141]. Many of these next-generation cationic-based polymers e.g., poly(β-amino esters) (PBAE) or poly[(2-dimethylamino) ethyl methacrylate] (pDMAEMA), have found to be beneficial for the formulation of mRNA, but also of other nucleotide moieties e.g., DNA and short interfering RNA (siRNA) [24,139]. However, efficient cationic polymers used for other nucleic acids delivery differ in binding strengths—hence, mRNA delivery based on them—might be sub-optimal. For instance, there are studies pointing out that single stranded mRNA binding to cationic polymers can be problematic since the binding is stronger than that observed with pDNA, ultimately affecting the mRNA release [142,143]. Several studies have led to the improvement of the in vitro mRNA transfection by further modifying novel successful synthetic polymeric materials. Such novel hyperbranced PBAES, poly(amino-co-ester) (PACE) and actuated PACE (aPACE) polymers have been developed and optimized as successful vehicles for mRNA delivery [12,144,145], while libraries containing potent polymers for mRNA delivery have been created to assess the structure–activity correlation and aid to further investigations [146]. Rapid advances in polymer chemistry should be exploited to discover and optimize more efficient polymers for enhanced mRNA encapsulation and transfection efficiency, while respecting safety issues and biocompatibility concerns.

#### 6.2.2. Lipid Based Vectors

The most common formulation strategy of mRNA delivery is nowadays the employment of lipid-based vectors. These systems are based on synthetic or natural lipids or lipid-like compounds (lipidoids) to form various delivery systems, such as liposomes (often referred as lipoplexes) or lipid nanoparticles (LNPs). Lipid nanoparticles (LNPs) have become the most widely used mRNA delivery carriers so far [147]. Lipoplexes, liposomes and lipid nanoparticles are structures that their properties allow for successful cellular delivery of RNA protecting it from RNase activity, while at the same time escaping endosomal capture [119]. Lipoplexes (complexes of liposomes and nucleic acids) consist of lipids positively charged (cationic lipids) which attract electrostatically the negatively charged mRNA [148]. Lipoplexes have the peculiarity of entrapping their nucleic acid payload on the surface of concentric sheet formations rather than their core [149]. Generally, a lipoplex contains a cationic lipid, e.g., dioctadecenyl-trimethylammoniumpropane (DOTMA) or dioleoyl-trimethylammonium-propane (DOTAP) and a neutralizer of the excessive cationic charge such as dioleoyl-phosphoethanolamine (DOPE). Moreover, they can be stabilized by the addition of sterols such as cholesterol [150]. Neutral lipids are also employed in order to improve transfection and limit the toxicity since pro inflammatory reactiors and adverse effects have been reported from their use [151,152]. Such liposomes have been successfully employed in order to deliver siRNA into mouse liver [153]. Moreover, Kranz et al. assessed the impact of lipoplex charge on RNA delivery in vivo. This study indicated that the ideal chemical composition of a lipoplex depends on the organ or tissue that is targeted [154].

Liposomes are self-assembling closed membrane structures, resulting from the dispersion of phospholipids in water [155,156]. Cationic liposomes form either single or multiple amphiphilic lipid bilayers that surround an aqueous core that carries the nucleic acid. The most frequently applied material for liposome structure is DOPE in conjunction with cholesterol [157]. Michel et al. (2017) managed to assess the stability, cytotoxicity and immunogenicity of liposomes in vitro. The results indicated that the transfection of liposomes didn’t affect cell viability and immune response [152]. Additionally, DOTAP/DOPE at a 1:1 ratio is effective for the in-vivo delivery and transfection of mRNA encoding an HIV1 antigen Gag [158]. In general, lipid-based vehicles share many advantages such as high cell-membrane permeability, high transfection efficiency, low toxicity, long time storage and easy mRNA encapsulation. However, liposomes have disadvantages; (1) liposomes are prone to oxidative reaction, (2) they have high complexity, (3) low loading capacity and poor potency due to their charged nature (charged cationic lipids that complex with the negatively charged mRNA) [159,160].

Surface modification of liposomes can overcome some of the limitations mentioned [161]. Lipid nanoparticles (LNPs) are the most advanced systems for mRNA transport. The morphology of lipid nanoparticles differs from a traditional liposome, in the sense that they tend to be mostly devoid of internal water content. Instead, their core is characterized by high electron density, and their payload, be it nucleic acid or other pharmaceutical agents, is encapsulated within inverted micelles that are formed by cationic ionizable lipids [80,160,162]. The development of ionizable cationic lipids has revolutionized the field of LNP production [160]. These lipids have the property of being pH reactive, being positively charged at acidic pH but mostly unchanged at the pH of blood. Therefore, the outer shell of an LNP consists of cationic ionizable lipids e.g., DOTMA (dioctadecenyl-trimethylammoniumpropane) containing an amino head group. This group is protonated at acidic pH, whereas its charge remains neutral at physiological pH, a behavior that can assist mRNA complexing, cellular entry, as well as release from the endosome [163]. These titratable aminolipids are often combined with other helper lipids and PEGylated lipids. Helper lipids such as DOPE, cholesterol or DSPC provide additional stability to NLP formulation, while polyethylene glycol (PEG)-conjugated lipids, such as DMG-PEG, optimizes pharmacokinetics and biodistribution of the product [164,165].

Recently, Xiong et al. (2020) utilized a nanoparticle containing dendrimers and lipids (DLNPs). In this structure, lipid components include DOPE, cholesterol and PEGylated BODIPY dyes (PBD) lipids. Contrary to DMG-PEG lipids, the PBD lipids have the advantage that they offer in-vivo and in-vitro imaging due to their photoactive core [166].

#### 6.2.3. Polymer-Lipid Hybrid Vectors

Lipid polymer hybrid vectors (LPNs) have been already used for the therapeutic delivery of siRNA in vitro and in vivo [167,168]. This hybrid formulation is thermodynamically favorable and promotes stability [169]. Common polymers utilized in LPN structure are polycaprolactone, PLGA and polylactic acid whereas the lipids used are DOTAP, 1,2-dilauroyl-sn-glycero-3-phosphocholine, 1,2-distearoyl-sn glycero-3-phosphocholine, lecithin, DSPE and PEG. Additionally, recent studies exhibit promising results for the delivery of mRNA. Zhao et al. assessed encapsulation and delivery of mRNA in a hybrid nanoparticle composed of a polymeric PLGA core in conjunction with N1,N3,N5-tris(2-aminoethyl)benzene-1,3,5-tricarboxamide (TT) derived lipid-like nanomaterial (TT3-LLN). The results of this study indicated an increase in delivery of mRNA in 3 three different human cell lines compared to other polymers, therefore enhancing the promising delivery role of such hybrid nanomaterials [170]. In addition, another study supports the efficient simultaneous delivery of siRNA and mRNA with lipid-polymer hybrid vehicles consisting of different lipidoids and polystyrenesulfonate (PSS) which is a polymer carrying a negative charge [171]. Along with these results, Kaczmarek et al. reported successful delivery of mRNA to the lungs in vivo, utilizing a co-formulation of poly(β-amino esters) (PBAEs) with lipid-polyethylene glycol (PEG) [172].

#### 6.2.4. Other Vectors

Positively charged peptides are also suitable candidates for mRNA delivery systems. Cell penetrating peptides (CPP) which enter into the cell through endocytosis-mediated translocation. Udhayakumar et al. was the first to use arginine rich peptide base mRNA nanocomplexes and supported the efficient delivery of mRNA encoding for various antigens to cells of the immune system by utilizing peptide containing the amphipathic RALA motif that can facilitate cellular entry [173]. The CPP RALA effectively delivered both eGFP and OVA mRNA and outperformed a conventional DOPE/DOTAP lipoplex vector. Furthermore, Van den Brand et al. analyzed the ability of a commercially available cell-penetrating peptide PepFect 14 (PF14) complexed with eGFP mRNA to “home in” on ovarian cancer cells that were xenografted in animals [174]. This nanoparticle formulation was able to successfully deliver eGFP/mRNA into cancer cells specifically, outperforming the commercially available lipofectamine Messenger/MAX.

In addition to arginine-rich motifs, histidine rich cell penetrating peptides (CPP) have been also analyzed. A recent study estimates that LAH4-L1 peptide is advantageous over RALA for in vitro delivery of mRNA to dendritic cells [175]. According to this study, LAH4-L1 vehicle activates innate immune system through pattern recognition receptors (PRRs). Recently, a polymer-peptide hybrid mRNA delivery structure has been introduced. This hybrid delivery system consists of both polymer Polylactic acid (PLA) and peptide RALA. The results indicated that this hybrid delivery system protected mRNA against degradation [176]. Although CPPs represent a promising and interesting delivery approach, they share some flaws such lack of toxicity studies and production cost.

Another promising delivery tool for therapeutic mRNAs could be exosomal delivery [177,178]. Given that exosomes play a pivotal role in mRNA transfer between cells and have membrane permeability properties, they could be an optimal delivery system for mRNAs. This procedure requires cells producing exosomes and purification of released exosomes [179]. Large scale production of exosomes is fraught with high costs and low efficiency. Furthermore, targeted distribution of exosomes containing therapeutic mRNA is another obstacle remaining to be tackled in order for this advanced delivery system to make the “bench to bedside” transition [180].

## 7. mRNA Stability and Modifications

While RNA is thermodynamically more stable in vitro than DNA, it is generally considered a more sensitive and unstable molecule [181]. A major liability factor is the ubiquitous presence of RNases (5′ exonucleases, endonucleases and 3′ exonucleases), which can rapidly hydrolyse mRNA molecules, dictating the necessity of a RNase-free environment from the research and development laboratory upwards to the final formulation facility of the finished product [182]. RNA also tends to be more chemically reactive than DNA as far as electrophilic additions, alkylations and oxidation is concerned, and it can display increased rate of hydrolysis in pH exceeding 6 [182,183]. Model calculations suggest that apart from pH value, hydrolysis is also a function of temperature and concentration of ions such as Mg^2+^ which are introduced in steps like IVT [61,184].

An illustrative example of how deviation to even one factor of the ones mentioned above can compromise mRNA integrity, arises from the rigorous study of SARS-CoV-2 mRNA vaccine stability: under strict “cold-chain” transport conditions a naked RNA molecule encoding the virus’s spike has a calculated half-life of about 900 days, stored in phosphate-buffer saline (pH 7.4) in complete absence of magnesium ions. The reported half-life is predicted to be diminished to just five days, if the product is exposed to temperatures of 37 °C [3]. Another factor determining stability, is RNA length which is calculated to be inversely correlated with stability against hydrolysis [3]. Attempts at fortification of mRNA stability employing lipid pharmacotechnic formulations are to be considered carefully: utilization of lipids whose cationic head group can lower the pKa of the 2-hydroxyl group residing on the ribose moiety of the mRNA are best avoided since even a 2-unit shift can significantly hasten mRNA hydrolysis [185,186].

The saga of mRNA instability continues in vivo where it is exposed to a physiological temperature of 37 °C, increased concentrations of magnesium ions as well as the enzymes of the host cell. Considering the above, effort has put in modifying the basic elements of mRNA and formulating it in ways that can putatively increase its stability.

### 7.1. Structural Modifications

**Figure 4:**
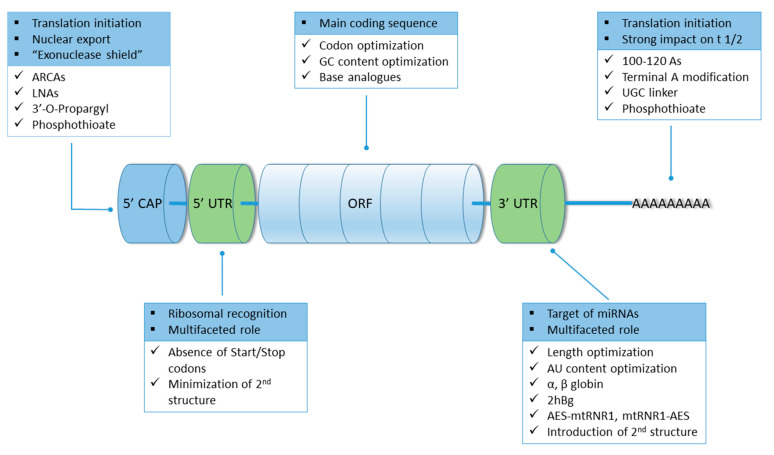
Structural elements of mRNA, their role and modifications impacting mRNA viability as a therapeutic. The main role of each element (5′ cap, 5′ and 3′ UTRs, ORF, Poly(A) tail) is given by a square bullet, whereas the modifications pertaining to its modification is given with checkmarks.

Five integral elements of mRNA are of pivotal importance for its therapeutical potential and have been the target of modifications: 5′ cap, 5′ and 3′ UTRs, ORF and the poly(A) tail. A summary of the modifications expanded on below can be seen in Figure 4.

#### 7.1.1. 5′ Cap

5′ cap is a structural element of cytosolic mRNA, absent from mitochondrial mRNA [187,188]. It features a modified guanosine methylated at the seventh position and bound to the rest of the molecule with a triphosphate group through an unusual 5′-5′ bond [38,189]. It plays a physiologically relevant role in splicing of the pre-mRNA and nuclear export of the mature mRNA through the nuclear pores [190]. Furthermore, it is implicated in translation initiation and cellular recognition of “self-molecules”, while at the same time acting as an “exonuclease shield”, safeguarding mRNA integrity [189]. The crucial role of the 5′ cap is highlighted by the highly orchestrated cellular process of decapping which leads to mRNA decay [191].

During mRNA manufacture, the 5′ capping performed at the stage of IVT carries the inherent danger of yielding a population of mixed mRNA species, some incorporating the 5′ cap in the correct orientation and some in reverse which leads to untranslatable mRNA [192]. A modification devised to surpass this hurdle lies in utilizing Anti-Reverse Cap Analogues (ARCA) technology, which ensures incorporation in the correct orientation. The simplest ARCA carries a modification where the 3′-OH of the m^7^ guanine moiety is substituted by a -OCH_3_ group, that yields a uniformly translatable population [189,193]. It has been shown that this modification results in mRNA species that display longer half-life in cultured cells, leading to an increased and sustained protein production [194,195], an effect also observable in cell-free lysates where doubled translation efficiency has been documented [196].

While the employment of ARCAs is thought to be the golden standard, alternative modifications have been proposed. A 3′-O-propargyl containing m^7^GpppG cap that is exclusively incorporated in the desired orientation resulted in 3-fold higher translation efficiency in HeLa cells compared to standard cap [197]. Modelling studies propose that this effect may be attributed to more stable complex formation with translation initiation factor eIF4E. Another strategy that can impact translational activity is to target the triphosphate m^7^G linkage moiety. A study has compared the activity of tetraphosphate dinucleotide cap analogues, such as m^7^Gp4m^7^G, to a regularly capped mRNA and found that the modification was also able to increase translation 3-fold [198]. Phosphothioate modifications of the 5′ cap appear to be promising as well, given that compared to standard cap they can yield a 3 to 5-fold increase in translation [199,200]. Lately, advancements in capping technology have yielded candidates that can outperform ARCAs. Such an example is the development of trinucleotide caps containing locked nucleic acid analogues which can increase translational efficiency of modified mRNAs by a factor of 5 compared to ARCAs as well as GAG cap in the mouse dendritic JAWSII cell line [201].

It should also be noted that, while not a part of the 5′ cap itself, the first nucleotide after it seems to play a role in mRNA stability. It is usually methylated on the 2-OH position of the ribose and in the case of adenosine a second methylation can take place at the N-6 position [202]. This specific double methylation, seems to exist in greater abundance in the cell and imbues mRNAs with longer half-lives due to probable increased DCP2-related decapping resistance, compared to the monomethylated counterparts [203,204,205].

5′ capping of mRNAs can be challenging when upscaling to industrial production. The price of the reagents, their availability in big quantities as well as behavior of the method can limit the available options. One of the capping options that has been demonstrated to be viable in large scale setting is the co-transcriptional incorporation of the cap, specifically a cap 1 by T7 RNA polymerase [206]. Cap 1 analogues, such as the ones provided commercially by the company Trilink, follow the general format of m7GpppNm, where the last nucleotide denoted as Nm is any nucleotide modified by a 2′O methylation. BioNTech COVID-19 vaccines that are currently on the market are co-transcriptionally capped using cap 1 analogues [28]. Another more complicated strategy is to deploy a post transcriptional capping. This strategy employs the addition of a Cap (m7GpppN, also known as cap 0) after IVT transcription using a Vaccinia virus capping enzyme, followed by its conversion to a Cap 1 structure by Vaccinia 2’O-methyltransferase [47]. This strategy was followed in the pre-clinical development of the Moderna vaccine. The intricacies introduced by capping procedures could probably be circumvented if an alternative approach to translation initiation is found to be viable. A promising candidate could be the inclusion of internal ribosomal entry site (IRES) sequence at the 5′ UTR region, which is capable of initiating translation in a cap independent manner [207]. IRES are found in both viral and eukaryotic genes and they are thought to provide a versatile translation initiation method should cap-depended translation is hindered to due factors such as stress [208]. The latter could even prove advantageous, should mRNA translation be desired in cells that have suppressed cap-depended translation. It has to be noted though, that currently cap-less IRES containing mRNAs, while being translatable, are still considered vulnerable towards degradation by exonucleases due the lack of a cap structure [48].

#### 7.1.2. 5′ and 3′ UTRs

5′ and 3′ UTRs are mRNA regulatory sequence elements implicated in recognition of the transcript by the ribosomes, facilitators of the interactions with the translational complex and targets of many mRNA binding proteins such as the ones regulation nuclear export of mature transcripts and subcellular localizations [209,210]. Considering their multifaceted role, the delineation of the effect of their structural specifics upon mRNA stability and translational efficiency has proven to be imperative in efficient mRNA design.

5′ UTRs can contain elements called upstream open reading frames (uORFs), are characterized by a conserved high GC content and feature the prominent presence of hairpins and other secondary structures [211]. Efficient mRNA design should take into account that Start, Stop and non-canonical Start codons present in uORF can hamper the main ORF translation [189]. Lengthwise, it has been found that increased length of 5′ UTR can be detrimental for mRNA eIF4F-depended translation so shorter sequences are generally preferred [212]. Length in conjunction with GC content can influence the mRNA fate through the formation of complex secondary structures characterized by highly negative ΔG. In theory, such formations can limit ribosomal and codon recognition factor accessibility to mRNA, negatively impacting translation [213]. Indeed, reduced secondary structure presence in the 5′ UTR region and 30 codons downstream, is correlated with increases in protein expression, although this is not always the case [214]. Highly structured 5′ UTRs such as the ones in dengue virus support efficient translation by means of increased polysome loading [215].

3′ UTRs are located immediately after the Stop codon and is known to serve a multifarious role in mRNA localization, stability and translation. They contain sites for a cornucopia of RNA binding proteins, target sequences for microRNAs, as well as transmission of genetic information to proteins via means of protein complex formation and post translational modifications [216,217].

When choosing a 3′ UTR for a therapeutic mRNA, length, sequence and secondary structure have to be taken into account. Longer 3′ UTRs tend to have shorter half-life, while overly short ones display reduced translational efficiency—hence, an optimal length has to be experimentally validated for each mRNA in question [218]. AU rich 3′ UTRs are subject to rapid degradation via association of the AU areas with RNA-binding proteins [219,220]. Given that such areas are commonly found in 3′ UTRs of proteins where prolonged expression could be detrimental to the cell, such as proto-oncogenes, cytokines and growth factors, AU enrichment can be employed when shorter acting mRNAs are desired [220].

Conversely if the objective is abundant expression, 3′ UTRs of ubiquitous protein mRNAs are preferred. Such an example is the common practice of employing 3′ UTRs of α- and β-globin to therapeutic mRNAs, which results in increases both in mRNA stability and translation efficiency [38]. Substituting a single β-globin with a two head-to-tail β-globin 3′ UTRs (2hBg) constitutes an improvement on the transcripts stability while further advancements have uncovered even more promising candidates. A fusion of the 3′ UTR of Aminoterminal Enhancer of Split (AES) and the 3′ UTR of mitochondrially encoded 12S rRNA (mtRNR1), either in the form of AES-mtRNR1 or mtRNR1-AES, has provided superior results compared to 2hBg in two applications: reprogramming of human fibroblasts to iPSC using Yamanaka factors and eliciting a robust immune response against gp70 in mice [218]. The improved performance observed might be attributable to a lower number of sites recognized by miRNAs and a higher total hybridization energy. An overall increased efficacy of mRNA might be improved by “mixing and matching” different sets of 5′ and 3′ UTRs, an increase attributable to facilitation of translation rather that increased stability [221]. Finally, 3′ UTRs in juxtaposition to 5′ UTRs might benefit from the presence of secondary structures, an observation that appears true for ORFs as well [214].

#### 7.1.3. Open Reading Frame

ORFs are the main coding sequences that are translated to peptides which reside between 5′ and 3′ UTRs. Widely employed modifications of the ORF regions include codon optimization and the use of base analogues [159,189].

Codon optimization entails the swapping of codons for synonymous ones, abiding to the codon bias of the organism in which the mRNA is to be introduced. The rationale behind such an optimization is that codons that correspond to relatively more abundant tRNA species speeds up translation rate yielding more protein before mRNA expires [222,223]. Expanding on the above, selection of optimal codons might also influence the mRNA molecule’s stability as well [224]. A complete optimization effort intergrades much more than simple selection of tRNA favored codons: it takes into account the GC content of the final transcript, as well as the strategic introduction of sub-optimality. GC rich mRNAs tend to have higher protein yields than AU rich counterparts since the latter tend to be more prone to post-transcriptional regulation through preferential accumulation to p-bodies, complication their fate within the cell [225]. On the other hand, a very high GC content might negatively affect overall performance through interference with secondary structure formation [189]. Translation of proteins with structurally sensitive areas susceptible to misfolding, could be aided by site-specific integration of sub-optimal codons. Such a modification slows down ribosomal processing long enough for efficient co-translational protein folding to take place, resulting in a functional peptide [222,226].

Use of base analogues can confer advantages both by ensuring proper protein folding through modulating, as above, production rate and by rendering mRNA invulnerable towards the cell’s innate immune response to foreign mRNAs [214,227]. Cellular recognition of exogenous mRNA is mediated through interaction of the offending molecule with endosomal Toll-like receptors (TLR) 3,7,8 and 12 which leads to its decay, when endogenous mRNA can escape such mechanisms due to the presence of post-translationally modified bases [228]. Common base analogues used to evade TLR recognition are pseudourine (Ψ), N1-methylpseudouridine (m^1^Ψ), 2-thiouridine (S^2^U), 5-methyluridine (m^5^U), 5-methylcytidine (m^5^C) and N^6^-methyladenosine (m^6^A) [159]. A rather successful strategy is uridine depletion, where immunogenic uridine content is minimized by increasing GC content where applicable or by substituting uridine with Ψ or m^1^Ψ [229,230]. As demonstrated by Kariko et al, substituting U for Ψ into mRNA results in a non-immunogenic product that displayed increased translational capacity both in vivo and in vitro, as the observed 9-fold in-vitro increase in translation highlights [231]. Similar, albeit less potent mRNAs have been achieved by incorporation of S^2^U, m^6^A, m^5^U and m^5^C while m^1^Ψ has proven to be more successful than Ψ in terms of non-immunogenicity and cytotoxicity [232]. Specifically concerning m^1^Ψ inclusion, it has been found that the combination of m^5^C and m^1^Ψ confers an enhanced capability of protein expression [232]. Combinations of S^2^U and m^5^C have given promising results in vivo: mRNAs coding for erythropoietin including 25% m^5^C and 25% S^2^U in their sequence, resulted in 5-fold higher levels of the target protein in mice [233]. Apart from methylated analogues, acetylated versions of bases such as N^4^-acetylcytidine (ac^4^C) have been employed showing an enhancement of mRNA translation both in-vivo and in-vitro, especially if they are present at the wobble position of the codon [234].

As in codon optimization, prudency is advised when considering the use of base analogues in mRNA as means to evade immunogenicity and increase translator efficiency. RNA modifications should be thought of more as dynamic regulators of the mRNA’s behavior, rather than unambiguous enhancers [235]. It appears that modified bases can exert a differential effect on the translation depending on their positioning in the codon. For example, in bacteria, the presence of m^6^A in the ORF and specifically on the first position appears to inhibit translation, a phenomenon also observed for Ψ in the third position of the codon [236]. This effect does not only affect translation efficiency, but it also carries the potential to “recode” a genome: Ψ presence in a stop codon can allow for read-through at least in yeast and bacteria [235]. Effects have also been observed in higher eukaryotes, where m^6^A can inhibit translation, especially if it is present multiple times or if it occupies the first position in a codon [237]. So far, no recoding effects have been observed in higher eukaryotes which can be attributed to the more robust control of protein synthesis. It has also been observed that not every mRNA responds positively to modifications thought to confer advantage via reduced immunogenicity and increased translation. For example, in HEK-239 cells, eGFP mRNAs decorated with multiple m^5^Cs or Ψs unexpectedly did not lead to higher protein levels [237]. Given the above, it is advisable that any time a new mRNA product is considered, modifications should be incrementally and combinatorially introduced in small scale experiments instead of resorting to a “copy and paste” approach of a previously successful mRNA modification motif. Such experiments can be performed in both eukaryotes and prokaryotes, utilizing methods that allow for site specific modification incorporation by means of RNA ligation [237,238].

#### 7.1.4. Poly(A) Tail

The presence of poly(A) tail on functional mRNA is essential and its length is crucial. The poly(A) tail can act as a facilitator in the formation of a circular closed loop state with the 5′ cap that inhibits deadenylation and promotes polysome formation [119,239]. The closed loop state is achieved through recruitment of polyadenosyl binding proteins (PABPs) by the tail that interact with eIF4 which recognizes and associates 5′ cap in order to initiate translation. The optimal length is a subject of debate; mammalian mRNAs may carry tails comprised of about 250 adenosines but it is considered that tails longer than 150 nucleotides have protruding parts that escape associating with PABPs. “Naked” mRNA poly(A) tails have a propensity to trigger the cytoplasmic deadenylation complex that can compromise mRNA longevity, hence more prudent sizes have been proposed for therapeutic applications [159,227]. A length of 100 to 120 nucleotides is thought to be ideal, increasing translation efficiency and at the same time contributing to uridine depletion by reducing the relative uridine content of mRNA [227,240].

Modifications of the poly(A) tail include use of adenosine analogues such as 8-azaadenosine, cordycepin, ribose-modified adenosines, fluorophore modified adenosines as well as the inclusion of phosphothioate bonds in the tail’s backbone [241,242]. Employment of analogues, specifically for the tail, is a terrain with at least two peculiarities. First of all, the general consensus is that the tail is intolerant of other bases than adenine, so the base used must strictly be an adenosine analogue. An exception to this “rule” constitutes the successful incorporation of a UGC linker in the poly(A) tail of the anti-SARS-CoV-2 Pfizer–BioNTech vaccine BNT162b2, where a 10-base pair segment was used to produce a A30 (10 bp UGC linker) A70 tail [243,244]. Moreover, the modified base must be incorporated at the very end of the tail and fluorophore incorporation might be inappropriate for therapeutical applications [241]. As far as phosphothioate groups are concerned, they seem to provide diminished susceptibility to mRNA degradation by 3′-deadenylase although this is not reflected on protein levels, which tend to remain the same [242].

## 8. Storage Considerations

It is common laboratory knowledge that long term mRNA storage can constitute a serious problem. Utilizing mRNA as a therapeutic makes the preservation of the final pharmacotechnic form, which is the mRNA plus the delivery vehicle, imperative since stability is required both in the chain of transport of the finalized product as well as during storage at the employment site. The most explored formulations as far as preservations issues are concerned, are the ones deployed currently in the BNT162b2 (Pfizer/BioNtech), mRNA-1273 (Moderna) and CVnCoV (Curevac) SARS-CoV-2 vaccines which are all mRNA Lipid nanoparticles (LNPs) [245].

When examining the storage conditions for mRNA-LNP formulations, it becomes apparent that as a rule of thumb they have ultra-low storage requirements; BNT162b2 can be stored at −60 to −80 for six months, −15 °C to −25 °C for two weeks and 2 °C to 8 °C for five days, mRNA-1273 can be preserved at −15 °C to −20 °C for six months and 2 °C to 8 °C for five days, while CVnCoV is an exception since it can be stored at 2 °C to 8 °C for up to three months [85]. Such requirements can be taxing from an economical perspective, if it is taken into account that the cold chain transport must abide to them, and temperature deviations can compromise the product’s integrity.

Two main vulnerabilities of the formulations discussed here are recognized: the LNP which can be prone to aggregation, leakage or fusion issues and the encapsulated mRNA which can be prone to degradation. The former can be easily addressed by the incorporation in the nanoparticle of stabilizing lipids and PEG, while the latter proves more difficult to tackle and it appears to be the major factor for product’s instability in non-frozen short-term storage [85,246]. It has been proposed that the main culprit undermining mRNA instability within the LNP, is the co-encapsulation of up to 24% water that can facilitate mRNA oxidation and hydrolysis of the phosphodiester backbone of the molecule [247]. In this case, the product could benefit from “tighter” encapsulation methods that will act exclusively towards water molecules, keeping the payload “dry” and intact. Recently, another vulnerability has been reported that concerns the interactions between lipids used in LNP production and the mRNA payload, yields a non-functional product [86]. The interaction pertains to tertiary amine containing lipids and can result in lipid-mRNA adduct formation that can escape common analytic procedures. As it appears, other kinds of lipids utilized in LNP formation do not seem to contribute to the lipid-mRNA adduct formation. The vulnerabilities mentioned above could be the starting point for an investigation on why the different mRNA products in the market have such highly variable storage condition requirements. Such an investigation could examine the impact on product stability of the different formulations taking into account both the lipids and the excipients, as well as the final nanostructure of the LNP produced.

Current approaches for preservation and storage include freezing and freeze-drying with the concomitant addition of cryoprotectants and lyoprotectants. Mannitol, sucrose and trehalose are commonly used cryoprotectants, usually added at a concentration of 5% allowing for in vivo delivery efficiency of 3 months if LNPs are stored in liquid nitrogen [72,248]. Increased concentrations of protectants, namely 10% sucrose, is being used in BNT162b and mRNA-1273 products to retain product viability at the more “cold-chain friendly” temperatures that they are preserved into [245]. Lyophilisation is also a viable, albeit more expensive procedure, that can yield a completely anhydrous product in powder form with extended self-life [249]. Lyoprotectants utilized can be the same as the cryoprotectants mentioned above as well as substances such as γ-cyclodextrin and hyaluronic acid [250]. Leaving lyophilisation aside, there are other presser vation technologies that, at least at laboratory scale, have given promising results such as spray and spray-freeze drying, supercritical fluid drying, foam drying and vacuum drying [246,251].

## 9. Conclusions

Faced with the global SARS-CoV-2 pandemic, the need for a versatile, scalable and rapid solution has led to the “weaponization” of a well-known, very promising but idiosyncratic candidate: mRNA. With the advent of successful and rapidly developable vaccines against SARS-CoV-2, the discussion on therapeutic use of mRNA is rekindled and expanded on a vast array of conditions not limited to infectious agents, but also cancers and medical entities that could benefit from protein replacement, genome editing and cellular reprogramming [1,38].

An mRNA approach towards a therapeutic problem has several major advantages compared to peptide biopharmaceuticals [19,38]. From a clinical point of view, mRNA therapeutics expel from the equation the sometimes undesirable and complicating factor of genomic integration of the peptide-producing sequence, while production-wise mRNA manufacture is easier to scale-up and comparatively less expensive. Contributor to the latter is the fact that a producing facility can manufacture a variety of mRNAs keeping the same workflow, equipment and running conditions, modifying only the initial plasmid sequence that codes for the new mRNA product.

The present review discusses some of the methods available for mRNA production both in laboratory and industrial setting, efforts undertaken to improve properties of mRNA such as stability, translational efficiency and lack of immunogenicity, as well as developments in vectors used to deliver the therapeutic payload into the recipient’s cells. The wake of an era of mRNA therapeutics, especially when the demand is of global scale, necessitates overcoming bottlenecks and hurdles in mRNA design and production. Amongst them is the availability and pricing of raw materials such as 5′ caps, enzymes and modified nucleotides, as well as issues of stability of the finalized formulation that is especially taxing, demanding an expensive cold-chain supply [19,117]. Hurdles like those can, in part, be overcome by research and implementation of technologies that could lower the amount of mRNA needed for the desired pharmacological response while keeping the same safety profile and improvements on storage procedures or vector formulations that could increase long-term stability [198] of the final product within a more reasonable and viable temperature range.

## Data Availability

Not applicable.
